# Supplementary material for: Multiscale modeling of HBV infection integrating intra- and intercellular viral propagation for analyzing extracellular viral markers
Source: bioRxiv. 2023 Jun 7:2023.06.06.543822. Preprint. [Version 1] doi: 10.1101/2023.06.06.543822 (PMC10274663; doi:10.1101/2023.06.06.543822)
Supplement: Supplement 1 [file NIHPP2023.06.06.543822v1-supplement-1.pdf]

bioRxiv preprint doi: https://doi.org/10.1101/2023.06.06.543822; this version posted June 7, 2023. The copyright holder for this preprint (which was not certified by peer review) is the author/funder, who has granted bioRxiv a license to display the preprint in perpetuity. It is made available under aCC-BY 4.0 International license.

LIST OF SUPPLEMENTARY MATERIALS

Figure S1 | Summary of HBV infection datasets

Figure S2 | Experiments using HBV-infected humanized mice

Figure S3 | Fitting of the mathematical model to the extracellular viral markers in peripheral blood of humanized mice treated with ETV or PEG IFN-α considering HBsAg production from iDNA

Table S1 | Estimated parameters for HBV infection in humanized mouse considering HBsAg production from iDNA

Table S2 | Fixed initial values for HBV infection in humanized mouse

Table S3 | Quantified results for cccDNA in HBV infected mouse

Supplementary Note 1 | Transformation to a system of ODEs from a PDE multiscale model

Supplementary Note 2 | Linearized equations under potent NAs treatment in humanized mouse

Supplementary Note 3 | Linearized equations under potent PEG IFN-α treatment in humanized mouse

Supplementary Information

Multiscale modeling of HBV infection integrating intra- and intercellular viral propagation for analyzing extracellular viral markers

Kosaku Kitagawa[1,†], Kwang Su Kim[1,2,†], Masashi Iwamoto[1,3,‡], Sanae Hayashi[4,‡], Hyeongki Park[1,‡], Takara Nishiyama[1], Naotoshi Nakamura[1], Yasuhisa Fujita[1], Shinji Nakaoka[5], Kazuyuki Aihara[6], Alan S. Perelson[7], Lena Allweiss[8,9], Maura Dandri[8,9], Koichi Watashi[3,10,11,#,*], Yasuhito Tanaka[4] and Shingo Iwami[1,11,12,13,14,15,#,*]

[1]interdisciplinary Biology Laboratory (iBLab), Division of Natural Science, Graduate School of Science, Nagoya University; Nagoya, Japan. [2]Department of Scientific Computing, Pukyong National University; Busan, South Korea. [3]Department of Virology II, National Institute of Infectious Diseases; Tokyo, Japan. [4]Department of Gastroenterology and Hepatology, Faculty of Life Sciences, Kumamoto University; Kumamoto, Japan. [5]Faculty of Advanced Life Science, Hokkaido University; Sapporo, Japan. [6]International Research Center for Neurointelligence, The University of Tokyo Institutes for Advanced Study, The University of Tokyo; Tokyo, Japan. [7]Theoretical Biology and Biophysics Group, Los Alamos National Laboratory; Los Alamos, USA. [8]Department of Internal Medicine, University Medical Center Hamburg-Eppendorf; Hamburg, Germany. [9]German Center for Infection Research (DZIF), Hamburg-Lübeck-Borstel-Riems partner sites; Germany. [10]Research Center for Drug and Vaccine Development, National Institute of Infectious Diseases; Tokyo, Japan. [11]Department of Applied Biological Sciences, Faculty of Science and Technology, Tokyo University of Sciences; Chiba, Japan. [12]Institute of Mathematics for Industry, Kyushu University; Fukuoka, Japan. [13]Institute for the Advanced Study of Human Biology (ASHBi), Kyoto University; Kyoto, Japan. [14]NEXT-Ganken Program, Japanese Foundation for Cancer Research (JFCR); Tokyo, Japan. [13]Interdisciplinary Theoretical and Mathematical Sciences (iTHEMS), RIKEN; Wako, Japan. [15]Science Groove Inc.; Fukuoka, Japan.

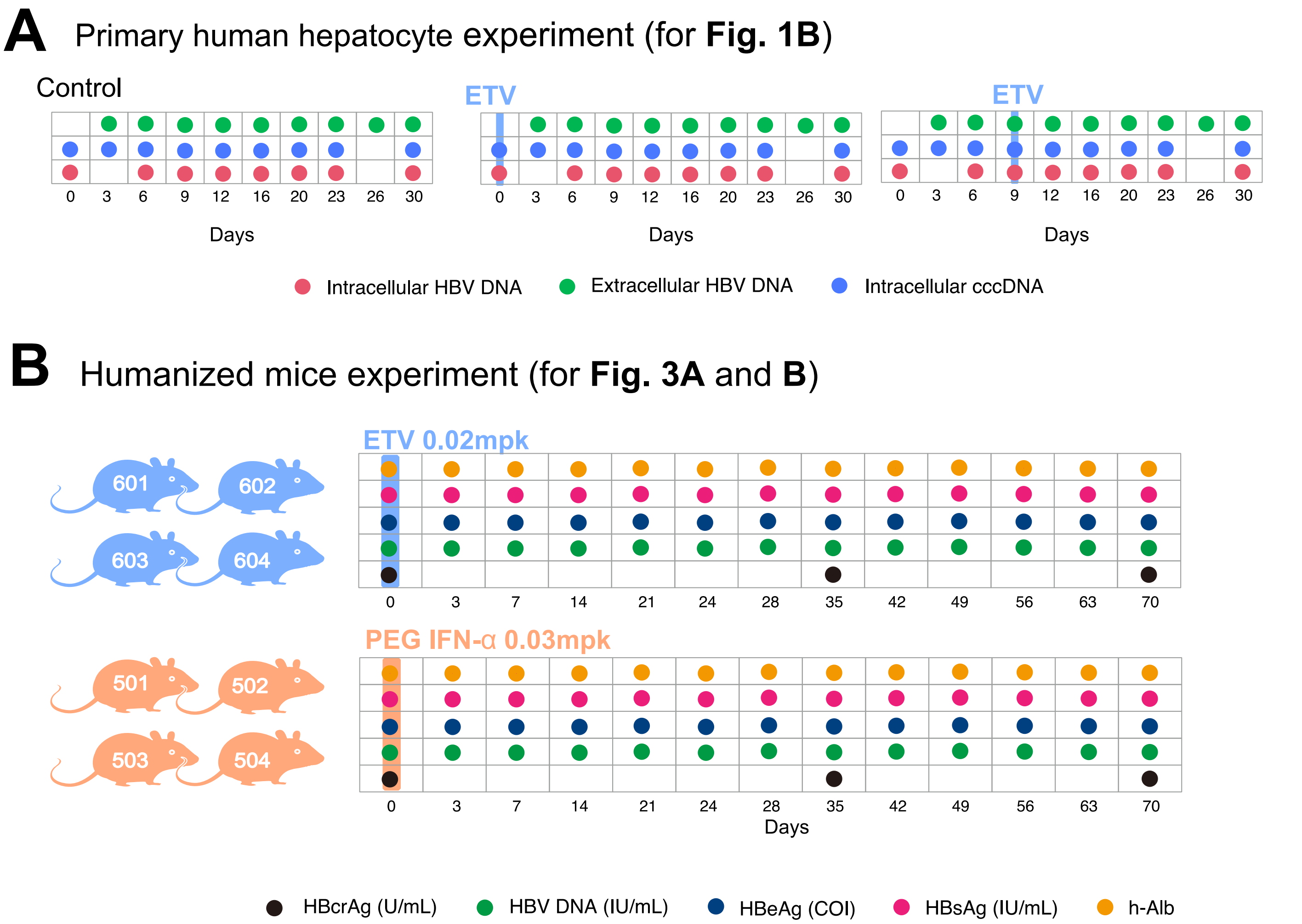

Figure S1. Summary of HBV infection datasets: Detailed data-sampling schedule for HBV-infected **(A)** primary human hepatocytes, and **(B)** humanized mice.

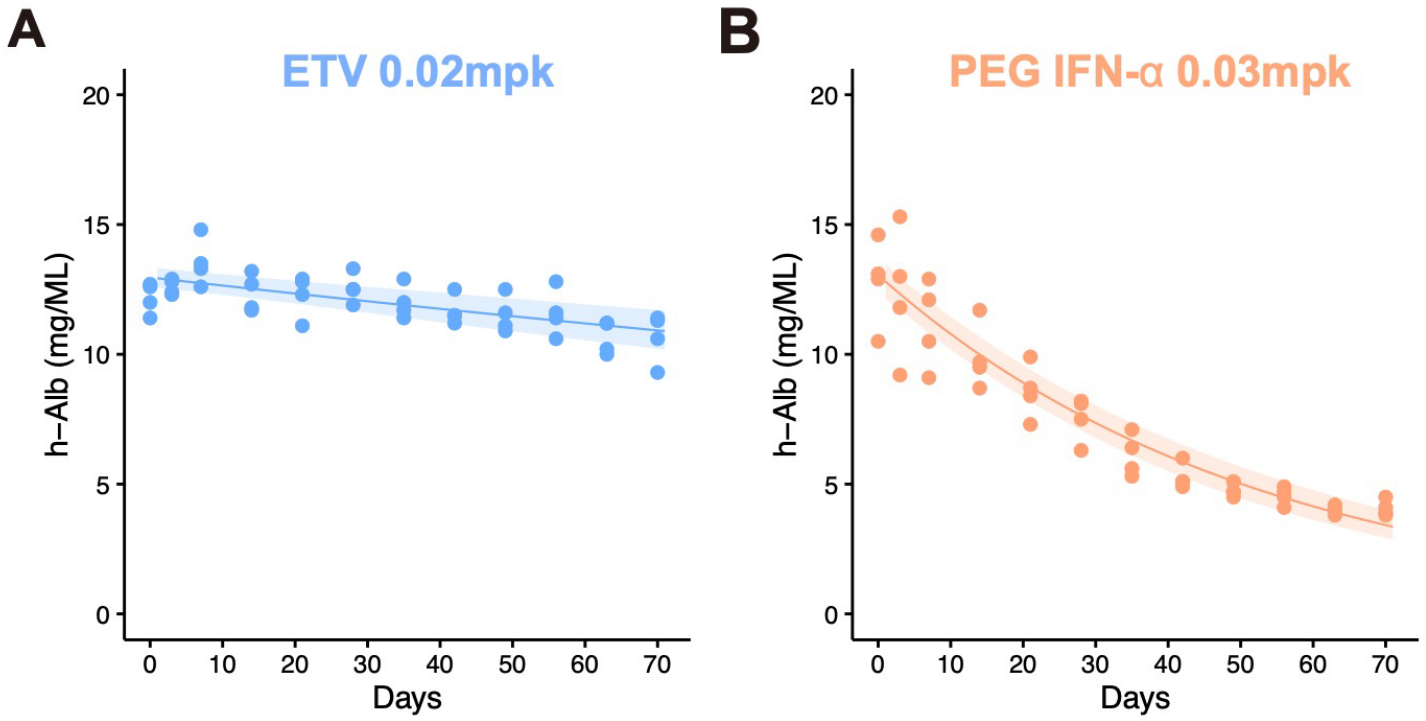

Figure S2. Experiments using HBV-infected humanized mice: Decay characteristics for h-Alb in peripheral blood of humanized mice treated with **(A)** ETV or **(B)** PEG IFN-α. The shaded regions correspond to 95% confidence intervals and the solid curves give the best-fit solution (mean) for a single decay model to the time-course dataset.

A x=0.5

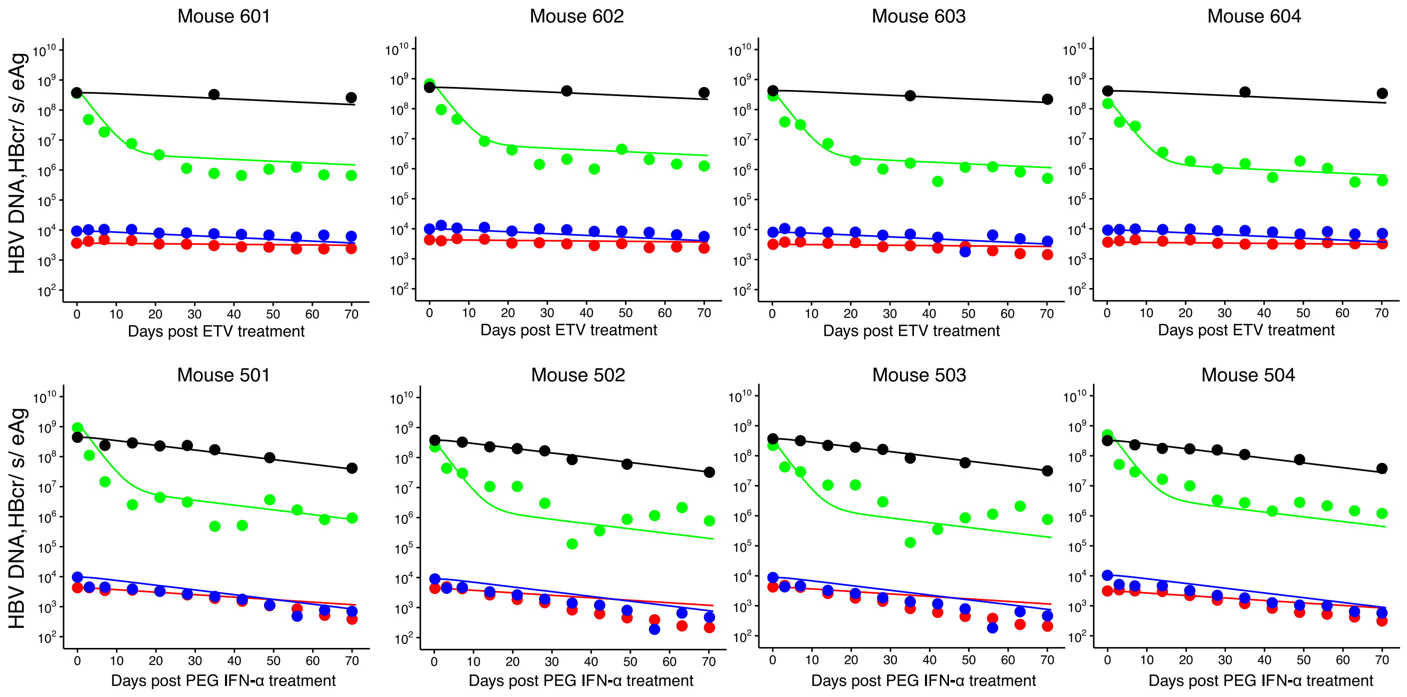

B x=0.8

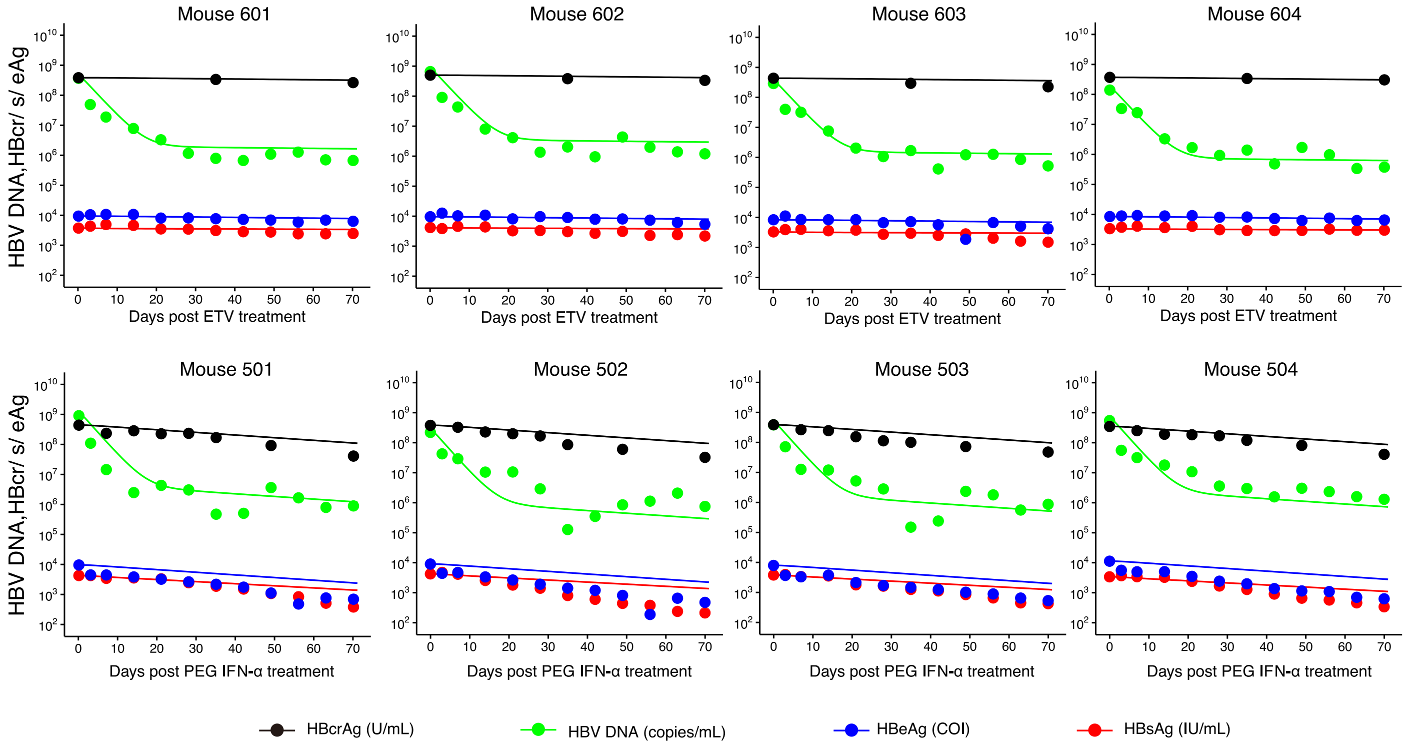

Figure S3. Dynamics of viral makers in HBV infected humanized mice: Fitting of the mathematical model to the extracellular viral markers in peripheral blood of humanized mice treated with ETV or PEG IFN-α considering HBsAg production from iDNA ($x = 0.5$ or 0.8).

Table S1. Estimated parameters for HBV infection in humanized mouse considering HBsAg production from iDNA

Parameters or variables	Symbol	Unit	Value
$\boldsymbol{x = 0.5}$			
Combined parameter†	$f\alpha$	-	5.4×10^{-3}
Inhibition rate of HBV DNA production	ε	-	9.7×10^{-1}
Decay rate of infected cells	δ	day^{-1}	2.4×10^{-3}
Decay rate of infected cells with IFN-α	δ_{IFN}	day^{-1}	1.9×10^{-2}
Degradation rate of cccDNA	d	day^{-1}	1.2×10^{-2}
Degradation rate of cccDNA with IFN-α	d_{IFN}	day^{-1}	1.7×10^{-2}
Release rate of intracellular HBV DNA	ρ	day^{-1}	3.8×10^{-1}
Residual sum of squares	--	-	23.123
$\boldsymbol{x = 0.8}$			
Combined parameter†	$f\alpha$	-	1.3×10^{-2}
Inhibition rate of HBV DNA production	ε	-	9.9×10^{-1}
Decay rate of infected cells	δ	day^{-1}	2.4×10^{-3}
Decay rate of infected cells with IFN-α	δ_{IFN}	day^{-1}	1.9×10^{-2}
Degradation rate of cccDNA	d	day^{-1}	4.6×10^{-4}
Degradation rate of cccDNA with IFN-α	d_{IFN}	day^{-1}	1.2×10^{-3}
Release rate of intracellular HBV DNA	ρ	day^{-1}	3.2×10^{-1}
Residual sum of squares	--	-	31.947

† Production rate of HBV DNA from cccDNA × Fraction of HBV DNA recycling for cccDNA

Table S2. Fixed initial values for HBV infection in humanized mouse

Variable	Symbol	Unit	Value
**ETV**			
Initial value for extracellular HBV DNA for Mouse 601	$V(0)$	copies/ml	3.68×10^9
Initial value for extracellular HBsAg for Mouse 601	$S(0)$	IU/ml	3.75×10^3
Initial value for extracellular HBeAg for Mouse 601	$E(0)$	COI	9.41×10^3
Initial value for extracellular HBcrAg for Mouse 601	$R(0)$	U/ml	3.85×10^9
Initial value for extracellular HBV DNA for Mouse 602	$V(0)$	copies/ml	6.53×10^9
Initial value for extracellular HBsAg for Mouse 602	$S(0)$	IU/ml	4.14×10^3
Initial value for extracellular HBeAg for Mouse 602	$E(0)$	COI	9.52×10^3
Initial value for extracellular HBcrAg for Mouse 602	$R(0)$	U/ml	4.97×10^9
Initial value for extracellular HBV DNA for Mouse 603	$V(0)$	copies/ml	2.82×10^9
Initial value for extracellular HBsAg for Mouse 603	$S(0)$	IU/ml	3.22×10^3
Initial value for extracellular HBeAg for Mouse 603	$E(0)$	COI	8.13×10^3
Initial value for extracellular HBcrAg for Mouse 603	$R(0)$	U/ml	4.25×10^9
Initial value for extracellular HBV DNA for Mouse 604	$V(0)$	copies/ml	1.48×10^9
Initial value for extracellular HBsAg for Mouse 604	$S(0)$	IU/ml	3.56×10^3
Initial value for extracellular HBeAg for Mouse 604	$E(0)$	COI	8.99×10^3
Initial value for extracellular HBcrAg for Mouse 604	$R(0)$	U/ml	3.92×10^9
**PEG IFN-α**			
Initial value for extracellular HBV DNA for Mouse 501	$V(0)$	copies/ml	9.26×10^9
Initial value for extracellular HBsAg for Mouse 501	$S(0)$	IU/ml	4.35×10^3
Initial value for extracellular HBeAg for Mouse 501	$E(0)$	COI	9.79×10^3
Initial value for extracellular HBcrAg for Mouse 501	$R(0)$	U/ml	4.49×10^9
Initial value for extracellular HBV DNA for Mouse 502	$V(0)$	copies/ml	2.29×10^9
Initial value for extracellular HBsAg for Mouse 502	$S(0)$	IU/ml	4.41×10^3
Initial value for extracellular HBeAg for Mouse 502	$E(0)$	COI	9.08×10^3
Initial value for extracellular HBcrAg for Mouse 502	$R(0)$	U/ml	3.81×10^9
Initial value for extracellular HBV DNA for Mouse 503	$V(0)$	copies/ml	3.66×10^9
Initial value for extracellular HBsAg for Mouse 503	$S(0)$	IU/ml	3.63×10^3
Initial value for extracellular HBeAg for Mouse 503	$E(0)$	COI	7.59×10^3
Initial value for extracellular HBcrAg for Mouse 503	$R(0)$	U/ml	3.69×10^9
Initial value for extracellular HBV DNA for Mouse 504	$V(0)$	copies/ml	5.03×10^9
Initial value for extracellular HBsAg for Mouse 504	$S(0)$	IU/ml	3.13×10^3
Initial value for extracellular HBeAg for Mouse 504	$E(0)$	COI	1.04×10^4
Initial value for extracellular HBcrAg for Mouse 504	$R(0)$	U/ml	3.22×10^9

Table S3. Quantified results for cccDNA in HBV infected mouse

Experimental group A	cccDNA† (band volume)	Average (band volume)	% of control
untreated control mouse A1	5.11×10^7	4.83×10^7	100
untreated control mouse A2	4.55×10^7	–	–
PEG IFN-α treated mouse A1	1.74×10^7	1.60×10^7	33
PEG IFN-α treated mouse A2	1.46×10^7	–	–
**Experimental group B**	**cccDNA (band volume)**	**Average (band volume)**	**% of control**
untreated control mouse B1	1.31×10^7	1.13×10^7	100
untreated control mouse B2	9.44×10^6	–	–
PEG IFN-α treated mouse B1	3.14×10^6	2.62×10^6	23
PEG IFN-α treated mouse B2	2.10×10^6	–	–

†cccDNA band volume was quantified from Southern blot data[1]. Briefly, mice infected with HBV at 12 weeks were treated with or without PEG IFN-α for 6 weeks, and then they were sacrificed. cccDNA levels were determined by Southern blot in Epicentre-based DNA extracts without proteinase K after PSD digestion. Experimental group A and B were performed as independent experiments.

Supplementary Note 1: Transformation to a system of ODEs from a PDE multiscale model

We here introduce a multiscale model using partial differential equations (PDEs) that couple intra-, inter- and extra-cellular virus dynamics for analyzing multiscale experimental data of HBV infection (c.f.[2]) (**Fig. 2**):

$$\frac{dT(t)}{dt} = s - d_T T(t) - \beta T(t)V(t), \tag{5}$$

$$\left(\frac{\partial}{\partial t} + \frac{\partial}{\partial a}\right) i(t,a) = -\delta i(t,a), \tag{6}$$

$$\frac{dV(t)}{dt} = (1-f)\rho \int_0^\infty D(a)i(t,a)da - \mu V(t), \tag{7}$$

$$\frac{dS(t)}{dt} = \pi_S \int_0^\infty C(a)i(t,a)da + s_i \int_0^\infty i(t,a)da - \sigma S(t), \tag{8}$$

$$\frac{dE(t)}{dt} = \pi_E \int_0^\infty C(a)i(t,a)da - \sigma E(t), \tag{9}$$

$$\frac{dR(t)}{dt} = \pi_R \int_0^\infty C(a)i(t,a)da - \sigma R(t), \tag{10}$$

$$\frac{dC(a)}{da} = f\rho D(a) - dC(a), \tag{11}$$

$$\frac{dD(a)}{da} = \alpha C(a) - \rho D(a). \tag{12}$$

As we recently reported,[3,4] the multiscale PDE model, Eqs.(5-12), can be transformed into a mathematically identical set of ordinary differential equations as follows. Using the method of characteristics with initial and boundary conditions of $i(t,a)$, we transform Eq. (6) into

$$i(t,a) = \begin{cases} e^{-\delta a} b(t-a) = e^{-\delta a}\beta T(t-a)V(t-a), & t > a, \\ e^{-\delta t} i_0(a-t), & t < a. \end{cases} \tag{S1}$$

Then, $I(t)$ is evaluated as follows:

$$I(t) = \int_0^t e^{-\delta a}\beta T(t-a)V(t-a)da + \int_t^\infty e^{-\delta t} i_0(a-t)da = \int_0^t e^{-\delta(t-a)}\beta T(a)V(a)da + \int_0^\infty e^{-\delta t} i_0(a)da.$$

Since $\frac{d}{dt}\int_0^t f(t,a)da = f(t,t) + \int_0^t \frac{\partial f(t,a)}{\partial t}da$, differentiating $I(t)$ with respect to time t, we obtain the following ODE:

$$\frac{dI(t)}{dt} = \beta T(t)V(t) - \delta I(t).$$

Also, we consider the total amount of cccDNA $CC(t)$ and the total amount of rcDNA $DD(t)$, defined by

$$CC(t) = CC(0) + \int_0^t C(a)i(t,a)da, \quad DD(t) = DD(0) + \int_0^t D(a)i(t,a)da,$$

respectively. Then we have

$$\frac{d}{dt}CC(t) = C(t)i(t,t) + \int_0^t C(a)\left(-\frac{\partial}{\partial a}i(t,a) - \delta i(t,a)\right)da = f\rho DD(t) - (d+\delta)CC(t),$$

$$\frac{d}{dt}DD(t) = D(t)i(t,t) + \int_0^t D(a)\left(-\frac{\partial}{\partial a}i(t,a) - \delta i(t,a)\right)da = \beta T(t)V(t) + \alpha CC(t) - (\rho+\delta)CC(t).$$

Therefore, the multiscale PDE model is described as the following equivalent system of ODEs:

$$\frac{dT(t)}{dt} = s - d_T T(t) - \beta T(t)V(t), \qquad (S2)$$

$$\frac{dI(t)}{dt} = \beta T(t)V(t) - \delta I(t), \qquad (S3)$$

$$\frac{dV(t)}{dt} = (1-f)\rho DD(t) - \mu V(t), \qquad (S4)$$

$$\frac{dS(t)}{dt} = \pi_S CC(t) + s_i I(t) - \sigma S(t), \qquad (S5)$$

$$\frac{dE(t)}{dt} = \pi_E CC(t) - \sigma E(t), \qquad (S6)$$

$$\frac{dR(t)}{dt} = \pi_R CC(t) - \sigma R(t), \qquad (S7)$$

$$\frac{dCC(t)}{dt} = f\rho DD(t) - (d+\delta)CC(t), \qquad (S8)$$

$$\frac{dDD(t)}{dt} = \alpha CC(t) - (\rho+\delta)CC(t) + \beta T(t)V(t). \qquad (S9)$$

Note that Eqs. (*S*2-*S*9) will be further simplified for the purpose of data analysis depending on the antiviral treatment assumed (see later).

Supplementary Note 2: Linearized equations under potent NAs treatment in humanized mouse

We assumed that NAs treatment is potent enough that intracellular HBV replications and *de novo* infections are negligible after treatment initiation[5-8], i.e., the antiviral effectiveness of NAs on intracellular HBV replications is assumed to be $0 < \varepsilon \leq 1$ and

$$i(t,a) = \begin{cases} 0 & t > a \\ i_0(a) & t < a \end{cases}.$$

Then Eqs. ($S2$-$S9$) can be simplified as follows:

$$\frac{dI(t)}{dt} = -\delta I(t), \qquad (S10)$$

$$\frac{dV(t)}{dt} = (1-f)\rho DD(t) - \mu V(t), \qquad (S11)$$

$$\frac{dS(t)}{dt} = \pi_S CC(t) + s_i I(t) - \sigma S(t), \qquad (S12)$$

$$\frac{dE(t)}{dt} = \pi_E CC(t) - \sigma E(t), \qquad (S13)$$

$$\frac{dR(t)}{dt} = \pi_R CC(t) - \sigma R(t), \qquad (S14)$$

$$\frac{dCC(t)}{dt} = f\rho DD(t) - (d+\delta) CC(t), \qquad (S15)$$

$$\frac{dDD(t)}{dt} = (1-\varepsilon)\alpha CC(t) - (\rho+\delta) CC(t). \qquad (S16)$$

Here we assume that all variables in Eqs. ($S2$-$S9$) are in steady state before treatment initiation[9], and particularly that the infected cells obtain a stable age distribution, i.e., $i_0(a) = \beta T(0)V(0)e^{-\delta a}$.

Since Eqs. ($S10$-$S16$) are a set of linear ODEs, we directly solve them, and find the following analytical solutions:

$$V(t) = V(0)\left(Ae^{(\lambda_1-\delta)t} + Be^{(\lambda_2-\delta)t} + (1-A-B)e^{-\mu t}\right), \qquad (S17)$$

$$S(t) = S(0)\left(Ce^{(\lambda_1-\delta)t} + De^{(\lambda_2-\delta)t} + Ee^{-\delta t} + (1-C-D-E)e^{-\sigma t}\right), \qquad (S18)$$

$$E(t) = E(0)(C'e^{(\lambda_1-\delta)t} + D'e^{(\lambda_2-\delta)t} + (1-C'-D')e^{-\sigma t}), \qquad (S19)$$

$$R(t) = R(0)(C'e^{(\lambda_1-\delta)t} + D'e^{(\lambda_2-\delta)t} + (1-C'-D')e^{-\sigma t}), \qquad (S20)$$

moreover, the total amount of cccDNA $CC(t)$ and the amount of cccDNA per infected cell $\tilde{C}(t) = CC(t)/I(t)$ are derived as follows:

$$CC(t) = CC(0)\left(Ze^{(\lambda_1-\delta)t} + (1-Z)e^{(\lambda_1-\delta)t}\right), \qquad (S21)$$

$$\tilde{C}(t) = \tilde{C}(0)\left(Ze^{\lambda_1 t} + (1-Z)e^{\lambda_1 t}\right), \qquad (S22)$$

where $A = \frac{-\{(\lambda_1+d+\delta)\lambda_2+\delta\rho\}\mu}{(\lambda_1-\delta+\mu)(\lambda_1-\lambda_2)(d+\delta)}$, $B = \frac{\{(\lambda_2+d+\delta)\lambda_1+\delta\rho\}\mu}{(\lambda_2-\delta+\mu)(\lambda_1-\lambda_2)(d+\delta)}$, $C = \frac{-(\lambda_2-\delta)\sigma p_i}{(\lambda_1-\delta+\sigma)(\lambda_1-\lambda_2)}$, $D = \frac{(\lambda_1-\delta)\sigma p_i}{(\lambda_2-\delta+\sigma)(\lambda_1-\lambda_2)}$, $E = \frac{\sigma(1-p_i)}{\sigma-\delta}$, $C' = \frac{-(\lambda_2-\delta)\sigma}{(\lambda_1-\delta+\sigma)(\lambda_1-\lambda_2)}$, $D' = \frac{(\lambda_1-\delta)\sigma}{(\lambda_2-\delta+\sigma)(\lambda_1-\lambda_2)}$, $Z = \frac{-\lambda_2+\delta}{\lambda_1-\lambda_2}$ and $\lambda_{1,2} = \frac{-(\rho+d)\pm\sqrt{(\rho-d)^2+4f(1-\varepsilon)\alpha\rho}}{2}$. Note

that $p_i = \frac{1-2x}{1-x}$ and x is the proportion of HBsAg produced from integrated DNA: $x = \frac{s_i I(0)}{\pi CC(0) + s_i I(0)}$.

Supplementary Note 3: Linearized equations under potent PEG IFN-α treatment in humanized mouse

We also assumed that PEG IFN-α treatment is potent enough that intracellular HBV replication and *de novo* infections are negligible after treatment initiation[6,7,10-12], i.e., the antiviral effect of PEG IFN-α on intracellular HBV replications is assumed to be $0 < \varepsilon \leq 1$ and

$$i(t,a) = \begin{cases} 0 & t > a \\ i_0(a) & t < a \end{cases}.$$

Then Eqs. ($S2$-$S9$) can be simplified to

$$\frac{dI(t)}{dt} = -\delta_{IFN} I(t), \qquad (S23)$$

$$\frac{dV(t)}{dt} = (1-f)\rho DD(t) - \mu V(t), \qquad (S24)$$

$$\frac{dS(t)}{dt} = \pi_S CC(t) + s_i I(t) - \sigma S(t), \qquad (S25)$$

$$\frac{dE(t)}{dt} = \pi_E CC(t) - \sigma E(t), \qquad (S26)$$

$$\frac{dR(t)}{dt} = \pi_R CC(t) - \sigma R(t), \qquad (S27)$$

$$\frac{dCC(t)}{dt} = f\rho DD(t) - (d_{IFN} + \delta_{IFN}) CC(t), \qquad (S28)$$

$$\frac{dDD(t)}{dt} = (1-\varepsilon)\alpha CC(t) - (\rho + \delta_{IFN}) CC(t). \qquad (S29)$$

In addition, it has been reported that PEG IFN-α induces interferon-stimulated genes (ISGs) and ISGs potentially degrade intracellular cccDNA. Therefore, we assumed PEG IFN-α increases the cccDNA degradation rate[13], i.e., d_{IFN} $(> d)$. Similarly, we assume that all variables in Eqs. ($S2$-$S9$) are in steady state before treatment initiation, and that the infected cells have obtained a stable age distribution, i.e., $i_0(a) = \beta T(0) V(0) e^{-\delta a}$. Because PEG IFN-α may enhance the decay rate of infected cells in HBV infection due to cytotoxic effects (but relatively mild), we assumed δ_{IFN} $(\geq \delta)$ in the data fitting (**Fig. 3AB** and **Fig. S3**). Solving Eqs. ($S21$-$S27$) we find

$$V(t) = V(0)\left(A_{IFN} e^{(\eta_1 - \delta_{IFN})t} + B_{IFN} e^{(\eta_2 - \delta_{IFN})t} + (1 - A_{IFN} - B_{IFN}) e^{-\mu t}\right), \qquad (S30)$$

$$S(t) = S(0)\left(C_{IFN} e^{(\eta_1 - \delta_{IFN})t} + D_{IFN} e^{(\eta_2 - \delta_{IFN})t} + E_{IFN}\, e^{-\delta_{IFN} t} + (1 - C_{IFN} - D_{IFN} - E_{IFN}) e^{-\sigma t}\right), \qquad (S31)$$

$$E(t) = E(0)\left(C'_{IFN} e^{(\eta_1 - \delta_{IFN})t} + D'_{IFN} e^{(\eta_2 - \delta_{IFN})t} + (1 - C'_{IFN} - D'_{IFN}) e^{-\sigma t}\right), \qquad (S32)$$

$$R(t) = R(0)\left(C'_{IFN} e^{(\eta_1 - \delta_{IFN})t} + D'_{IFN} e^{(\eta_2 - \delta_{IFN})t} + (1 - C'_{IFN} - D'_{IFN}) e^{-\sigma t}\right), \qquad (S33)$$

moreover, the total amount of cccDNA $CC(t)$ and the amount of cccDNA per infected cell $\tilde{C}(t) =$

$CC(t)/I(t)$ are derived as follows

$$CC(t) = CC(0)\left(Z_{IFN}e^{(\eta_1-\delta_{IFN})t} + (1-Z_{IFN})e^{(\eta_2-\delta_{IFN})t}\right), \qquad (S34)$$

$$\tilde{C}(t) = \tilde{C}(0)(Z_{IFN}e^{\eta_1 t} + (1-Z_{IFN})e^{\eta_2 t}), \qquad (S35)$$

where $A_{IFN} = \frac{-\{(\eta_1+d+\delta)\eta_2+(d-d_{IFN}+\delta)\rho\}\mu}{(\eta_1-\delta_{IFN}+\mu)(\eta_1-\eta_2)(d+\delta)}$, $B_{IFN} = \frac{\{(\eta_2+d+\delta)\eta_1+(d-d_{IFN}+\delta)\rho\}\mu}{(\eta_2-\delta_{IFN}+\mu)(\eta_1-\eta_2)(d+\delta)}$, $C_{IFN} = \frac{-(\eta_2-d+d_{IFN}-\delta)\sigma p_i}{(\eta_1-\delta_{IFN}+\sigma)(\eta_1-\eta_2)}$, $D_{IFN} = \frac{(\eta_1-d+d_{IFN}-\delta)\sigma p_i}{(\eta_2-\delta_{IFN}+\sigma)(\eta_1-\eta_2)}$, $E_{IFN} = \frac{\sigma(1-p_i)}{\sigma-\delta_{IFN}}$, $C'_{IFN} = \frac{-(\eta_2-d+d_{IFN}-\delta)\sigma}{(\eta_1-\delta_{IFN}+\sigma)(\eta_1-\eta_2)}$, $D'_{IFN} = \frac{(\eta_1-d+d_{IFN}-\delta)\sigma}{(\eta_2-\delta_{IFN}+\sigma)(\eta_1-\eta_2)}$, $Z_{IFN} = \frac{-\eta_2+d-d_{IFN}+\delta}{\eta_1-\eta_2}$ and $\eta_{1,2} = \frac{-(d_{IFN}+\rho)\pm\sqrt{(d_{IFN}-\rho)^2+4f(1-\varepsilon)\alpha\rho}}{2}$. Note that $p_i = \frac{1-2x}{1-x}$ and x is the proportion of HBsAg produced from integrated DNA: $x = \frac{s_i I(0)}{\pi CC(0)+s_i I(0)}$.

References

1. Allweiss, L., *et al.* Therapeutic shutdown of HBV transcripts promotes reappearance of the SMC5/6 complex and silencing of the viral genome in vivo. *Gut* (2021).
2. Iwanami, S., *et al.* Should a viral genome stay in the host cell or leave? A quantitative dynamics study of how hepatitis C virus deals with this dilemma. *PLoS Biol* **18**, e3000562 (2020).
3. Kitagawa, K., *et al.* Mathematical Analysis of a Transformed ODE from a PDE Multiscale Model of Hepatitis C Virus Infection. *Bull Math Biol* **81**, 1427-1441 (2019).
4. Kitagawa, K., Nakaoka, S., Asai, Y., Watashi, K. & Iwami, S. A PDE multiscale model of hepatitis C virus infection can be transformed to a system of ODEs. *J Theor Biol* **448**, 80-85 (2018).
5. Alonso, S., *et al.* Upcoming pharmacological developments in chronic hepatitis B: can we glimpse a cure on the horizon? *BMC Gastroenterol* **17**, 168 (2017).
6. Fatehi, F., Bingham, R.J., Stockley, P.G. & Twarock, R. An age-structured model of hepatitis B viral infection highlights the potential of different therapeutic strategies. *Sci Rep* **12**, 1252 (2022).
7. Goyal, A., Liao, L.E. & Perelson, A.S. Within-host mathematical models of hepatitis B virus infection: Past, present, and future. *Curr Opin Syst Biol* **18**, 27-35 (2019).
8. Wolters, L.M., Hansen, B.E., Niesters, H.G., DeHertogh, D. & de Man, R.A. Viral dynamics during and after entecavir therapy in patients with chronic hepatitis B. *J Hepatol* **37**, 137-144 (2002).
9. Neumann, A.U., *et al.* Hepatitis C viral dynamics in vivo and the antiviral efficacy of interferon-alpha therapy. *Science* **282**, 103-107 (1998).
10. Colombatto, P., *et al.* A multiphase model of the dynamics of HBV infection in HBeAg-negative patients during pegylated interferon-alpha2a, lamivudine and combination therapy. *Antivir Ther* **11**, 197-212 (2006).
11. Ribeiro, R.M., *et al.* Hepatitis B virus kinetics under antiviral therapy sheds light on differences in hepatitis B e antigen positive and negative infections. *J Infect Dis* **202**, 1309-1318 (2010).
12. Reinharz, V., *et al.* Understanding Hepatitis B Virus Dynamics and the Antiviral Effect of Interferon Alpha Treatment in Humanized Chimeric Mice. *J Virol* **95**, e0049220 (2021).
13. Lucifora, J., *et al.* Specific and nonhepatotoxic degradation of nuclear hepatitis B virus cccDNA. *Science* **343**, 1221-1228 (2014).
